# A Type III Effector NleF from EHEC Inhibits Epithelial Inflammatory Cell Death by Targeting Caspase-4

**DOI:** 10.1155/2017/4101745

**Published:** 2017-05-16

**Authors:** Ting Song, Kaiwu Li, Wei Zhou, Jing Zhou, Yuan Jin, Hongmei Dai, Tingting Xu, Mingda Hu, Hongguang Ren, Junjie Yue, Long Liang

**Affiliations:** ^1^State Key Laboratory of Pathogen and Biosecurity, Beijing Institute of Biotechnology, Beijing 100071, China; ^2^Institute of Health Sciences, Anhui University, Hefei, Anhui 230601, China

## Abstract

Enterohemorrhagic* E. coli* (EHEC) is a highly pathogenic bacterial strain capable of inducing severe gastrointestinal disease. Here, we show that EHEC uses the T3SS effector NleF to counteract the host inflammatory response by dampening caspase-4-mediated inflammatory epithelial cell death and by preventing the production of IL-1*β*. The other two inflammatory caspases, caspase-1 and caspase-5, are not involved in EHEC Δ*nleF*-induced inflammatory cell death. We found that NleF not only interrupted the heterodimerization of caspase-4-p19 and caspase-4-p10, but also inhibited the interaction of caspase-1 and caspase-4. The last four amino acids of the NleF carboxy terminus are essential in inhibiting caspase-4-dependent inflammatory cell death.

## 1. Introduction

The intestinal epithelium is an essential component of the host immune defense which provides a physical barrier between the body and the outside environment [1]. The innate immune response represents the first active line of defense against enteric pathogens [2]. Once activated, the innate immune response rapidly mediates the induction of cell death in infected host cells, the secretion of proinflammatory cytokines, and the control of invading pathogens [3]. Host cell death is a critical immune defense mechanism in response to microbial infection that sacrifices infected cells for the benefit of the remaining cells.

Although the modalities of host cell death induced upon bacterial infection vary among different pathogens, host cell type, or infectious stage, the following three types of host cell death are widely used as targets for bacterial pathogens to enhance pathogenesis: apoptosis, necrosis, and pyroptosis [4]. Apoptosis is a type of noninflammatory programmed cell death that is morphologically characterized by cell shrinkage, membrane blebbing, mitochondrial permeability, and DNA fragmentation. Bacteria are retained within apoptotic bodies and engulfed by phagocytic cells during this process [5]. Necrosis is triggered by ROS production or danger signals that are induced upon bacterial infection or physical damage. In necrosis, cell membrane rapidly ruptures and the cellular contents are released, accompanying caspase-independent inflammation [6]. Pyroptosis is also known as inflammatory cell death, which is coordinated by inflammasome-mediated caspases activation and accompanied by DNA fragmentation, membrane rupture, and the release of proinflammatory cytokines, including IL-1*β* and IL-18. Human caspase-1, caspase-4, and caspase-5 are critical mediators in the pyroptosis process [7].

Invasion of the mucosal epithelium by bacterial pathogens often triggers various innate immune responses such as proinflammatory signaling, the restriction of intracellular bacterial growth, and the demise of infected cells [8]. Among these immune responses, inflammatory cell death is a major innate defense mechanism that efficiently eliminates invaders and releases alarm signals for activating the host innate immune system [9]. One recent study reported that* S. typhimurium*-infected intestinal epithelial cells undergo pyroptosis and release IL-18. The authors showed that decreased rates of pyroptotic cell death could extrude infected cells from the polarized epithelium, accounting for increased pathogen burdens [10]. However, many enteric pathogens, such as enteropathogenic* Escherichia coli* (EPEC),* Shigella*, and* Salmonella*, use various strategies to manipulate the host cell death and to enhance their replication and survival. As typical pathogenic Gram-negative bacteria, these pathogens utilize a syringe-like T3SS to translocate virulence proteins (effectors) into mammalian host cells, which facilitate bacterial colonization and immune evasion and regulate host cell death and inflammatory response [11, 12]. For example, EPEC counteracts the apoptotic cell death by translocating NleH1 and NleH2, which block caspase-3 activation, nuclear condensation, and membrane blebbing [13].* Shigella* infection induces acute inflammatory epithelial cell death, which is counteracted by the effector OspC3. Infection of guinea pigs with* Shigella ospC3*-deficient mutant results in enhanced pyroptosis and decreased bacterial burdens [14].* Salmonella* inhibits apoptosis and activates prosurvival signals, dependent on the effectors AvrA and SopB, respectively [15]. These T3SS effectors have emerged as important mediators of virulence through activities; they regulate cell survival and death in a coordinated spatial and temporal manner [16].

As the major causative pathogens of food poisoning worldwide, EPEC and EHEC use various T3SS effectors to regulate the host innate immune response. These effectors have highly specific and diverse functions that interfere with a range of innate signaling pathways, including NF-*κ*B and MAPK activation [9]. NleB functions as a translocated N-acetyl-D-glucosamine (O-GlcNAc) transferase, which mediates GAPDH O-GlcNAcylation, thus inhibiting both TRAF2 activation and downstream NF-*κ*B signaling [17, 18]. NleC and NleD are Zn-dependent proteases that specifically clip and inactivate RelA (p65) and JNK, respectively, thus blocking NF-*κ*B and AP-1 activation [19, 20]. NleE harbors an methyltransferase activity, which specifically modifies TAB2 and TAB3, thus diminishing their ubiquitin-chain binding activity and dampening host NF-*κ*B signaling [21]. NleF is a potent inhibitor of mammalian caspase-4/11 and thus prevents IL-18 secretion in vitro, and it blocks caspase-11-IL-18-mediated neutrophil influx during infection [22]. However, no EHEC effectors have been demonstrated to directly inhibit the host inflammatory cell death.

Here, we report an offense-defense mechanism that occurs during EHEC O:157 infection of the epithelium and show that NleF inhibits inflammatory cell death mediated by caspase-4. In this process, NleF prevents the production of IL-1*β* and interrupts the heterodimerization of caspase-4-p19 and caspase-4-p10. Our findings provide the first example of EHEC-mediated suppression of inflammatory cell death in which NleF plays a novel role in controlling the host immune response through targeting of caspase-4.

## 2. Materials and Methods

### 2.1. Bacterial Strains

EHEC EDL933 (WT* E. coli* O157: H7) was previously described [23]. Nonpolar deletion mutant of* nleF* in* E. coli* O157:H7 EDL933 was generated by *λ* red-mediated mutagenesis [24]. Deletions replaced* nleF* codons with a* kanamycin* resistance marker in* E. coli*. Primers Z6020-K1 and Z6020-K2 and plasmid pKD4 were used to generate the deletion cassette, which was transformed into EHEC O157:H7 EDL933 strain via the PKD46 plasmid by electroporation. The replacement of the* nleF* gene by* kanamycin* resistance gene was confirmed by PCR using primers Z6020F and Z6020R. Plasmid pcP20 was used to remove the* kanamycin* resistance marker [24]. The growth kinetics of the* nleF* mutants did not differ from the parental strains.

The* nleF*-complemented strain (Δ*nleF/nleF*) was constructed as follows: the entire coding region of* nleF* was amplified from EHEC chromosomal DNA by PCR with His-NleF-1 and His-NleF-2 primers containing restriction sites for* Nde* I and* EcoR* I, respectively. The purified PCR products were digested with* Nde* I and* EcoR* I and cloned into the corresponding sites of IPTG-inducible expression vector pET-24a(+). The plasmid that contains* nleF* gene was transformed by electroporation into the donor strain Δ*nleF*, creating the strain with* nleF* complementation.

The primer sequences used for PCR were as follows:

Z6020-K1: TGTTAAGGGGGTTTTGATATGTTACCAACAAGTGGTTCTTCATGTAGGCTGGAGCTGCTTCG
Z6020-K2: AACTCACAGACCTCTAATCATCCACATTGTAAAGATCCTTTCATATGAATATCCTCCTTAG
  Z6020F: CGGGATCCTCAATGTTGGTGTGAATG
  Z6020R: GACGGACGAGTCAGTAAAAAAAGT
  His-NleF-1: CCATATGATGTTACCAACAAGTGGTTCTTC
  His-NleF-2: CGAATTCTCCACATTGTAAAGATCCTTTG

### 2.2. Cell Culture and Transfection

HT29 (human colon adenocarcinoma) and 293T (human embryonic kidney) cells were cultured in Dulbecco's modified Eagle's medium (DMEM, Gibco) supplemented with 10% heat-inactivated fetal bovine serum (FBS, Gibco), 2 mM L-glutamine, 100 U/mL penicillin, and 100 mg/mL streptomycin. Transient transfections were performed with Lipofectamine 2000 (Invitrogen) according to the manufacturer's instructions.

### 2.3. Antibodies, Reagents, Immunoprecipitation, and Immunoblotting

Anti-Flag (Sigma-Aldrich), anti-Myc (Santa Cruz), anti-*α*-tubulin (Sigma-Aldrich), anti-His (Origene), anti-GST (Origene), and anti-HA (Origene) mouse monoclonal antibodies as well as anti-IL-1*β* (Santa Cruz) rabbit polyclonal antibodies were used as primary antibodies for immunoblotting and immunoprecipitation. HRP-conjugated goat anti-rabbit (ZSGB-BIO) or anti-mouse IgG (ZSGB-BIO) antibodies were used as secondary antibodies for immunoblotting.

Immunoprecipitation was performed using IP buffer (1% Nonidet P-40, 50 mM Tris-HCl [pH 7.5], 150 mM NaCl, and Complete™ protease inhibitor cocktail-EDTA (Roche)). Whole cell extracts were prepared after transfection and incubated with indicated antibodies together with Protein A/G beads (Roche) overnight. Beads were then washed 4 times with IP buffer, and immunoprecipitates were eluted with SDS loading buffer (TransGen Biotech) and resolved in SDS-PAGE gels. The proteins were transferred to PVDF membrane (Bio-Rad) and further incubated with the indicated antibodies. The antigen-antibody complexes were visualized by the Immubilon™ chemiluminescent detection kit (Millipore).

### 2.4. GST Pull-Down Assay

For GST fusion proteins, the* E. coli* BL21 (DE3) strain harboring pGEX-2TK derivatives was cultured in Luria broth (LB) supplemented with ampicillin (100 *μ*g/mL) at 37°C until OD_600_ = 0.4. Protein expression was induced by adding 1 mM isopropyl-1-thio-*β*-D-galactopyranoside (IPTG), and then the cultures were incubated for 3.5 h at 30°C. Bacteria were resuspended in NETN buffer (20 mM Tris-HCl, 100 mM NaCl, 1 mM EDTA, and 0.5% NP40) disrupted by sonication treatment. The GST fusion proteins were purified with Glutathione Sepharose 4B (GE Healthcare) according to the manufacturer's protocol.

BL21 (DE3) cells expressing His-tagged proteins were lysed with lysis buffer (1% Triton X-100, 50 mM Tris-HCl [pH 7.5], 150 mM NaCl, 1 *μ*M 2-mercaptoethanol, 1 *μ*M 4-(2-aminoethyl) benzenesulfonyl fluoride, and 1 mg/mL lysozyme) and centrifuged at 14,000 ×g at 4°C for 20 min. The cleared lysates were mixed with GST derivatives bound to Glutathione Sepharose 4B beads for 2 h at 4°C. Then, the beads were washed four times with lysis buffer, and the bound proteins were analyzed by immunoblotting using the indicated antibodies.

### 2.5. Competition Assay

His-tagged protein was purified as previously described [23]. Flag-tagged recombinant protein expressed in 293T cells was immunoprecipitated through anti-Flag antibody conjugated agarose gel (Sigma-Aldrich). Proteins were eluted off beads using 3×Flag peptide (Sigma-Aldrich). Various concentrations (0.3, 0.6, 1.2, and 2.5 *μ*M) of purified Flag-NleF or Flag-NleF (Δ4aa) protein was preincubated with purified His-casp4-p10 (His-casp1-p10 or His-casp5-p10) protein (1.5 *μ*M) diluted in wash buffer (PBS containing 0.5% Triton X-100 and 10 *μ*M Z-VAD) for 2 h at 4°C. The mixtures were incubated with GST-casp4-p19 (GST-casp1-p20 or GST-casp5-p20) beads for 2 h at 4°C. Then, the beads were washed with wash buffer, and the bound proteins were analyzed by immunoblotting using the indicated antibodies.

### 2.6. Bacterial Infections

For infection, EHEC derivatives were cultured in LB at 37°C for 16 h with appropriate antibiotics. Overnight LB cultures were primed for infection by 1 : 100 dilution into serum-free and antibiotic-free DMEM, followed by further incubation for about 3 h at 37°C until OD_600_ = 1.0. HT29 cells were infected with the different EHEC strains at a multiplicity of infection (MOI) of 100 for the specified time points. The infection was initiated by centrifuging the plate at 700 ×g for 10 min. Following 1 hr incubation at 37°C, the plates were washed once with PBS and transferred into fresh medium. Cells were further incubated and samples (cells or cell culture medium) were harvested for analysis at time indicated.

### 2.7. Giemsa Staining of* E. coli* O157:H7 on HT29 Cells

After infection, HT29 cells were fixed with 4% paraformaldehyde (PFA) for 15 min and washed three times with PBS. Then, the cells were subjected to Giemsa staining for 45 min and washed three times with PBS.

### 2.8. IL-1*β* ELISA and Cytotoxicity Assay

Cells were plated, cultured for 24 h, and then infected with the different EHEC strains as described above. Aliquots of cellular supernatants were harvested at the indicated times after infection and transferred into 96-well plates (round bottom). Then, the supernatants were centrifuged at 200 ×g for 5 min, transferred into another 96-well plate (flat bottom), and subjected to the IL-1*β* ELISA using the Human IL-1*β* ELISA kit (R&D Systems) or the cytotoxicity assay using CytoTox 96® Non-Radioactive Cytotoxicity Assay (Promega) according to the manufacturer's instructions. Each sample was tested in triplicate.

### 2.9. DNA Manipulation

The* nleF* gene was PCR-amplified from EDL933 and cloned into the pGEX-2TK, pCMV-Myc, pCMV-HA, pET-24a (+), or pCDNA3-Flag vectors. To construct the NleF (Δ4aa) truncated mutant, the Δ4aa sequence was introduced into pCDNA3-Flag using a QuickChange™ site-directed mutagenesis kit (Stratagene). cDNAs for human* CASP1*,* CASP4*, and* CASP5* were obtained from Sino Biological Inc. These genes were cloned into pCDNA3-Flag or pCMV-Myc. casp4-p19, casp4-p10, casp1-p20, casp1-p10, casp5-p20, and casp5-p10 were PCR-amplified using pCDNA3-Flag-casp4, pCDNA3-Flag-casp1, or pCDNA3-Flag-casp5 as template and cloned into the pGEX-2TK and pET-24a (+) vectors.

### 2.10. siRNA

For siRNA knockdown, HT29 cells were cultured in 6-well plates at 40% confluency at the time of transfection. siRNA transfection was performed using the INTERFERin reagent (Polyplus Transfection) by following the manufacturer's instructions. The siRNA sequences were as follows: human casp1, 5′-GGUUCGAUUUUCAUUUGA G-3′ and 5′-CUCAAAUGAAAAUCGAACC-3′; human casp4, 5′-UCUACACUAUAGUCCAGACCC-3′ and 5′-GUCUGGACUAUAGUGUAGAUG-3′; human casp5, 5′-AUAGAACGAGCAACCUUGAC-3′ and 5′-GUCAAGGUUGCUCGUUCUAU-3′.

### 2.11. Statistical Analysis

Analyses were done with the statistical software SAS/STAT. Data analysis over time was undertaken by repeated-measures analysis with SAS/STAT. Differences were considered statistically significant if the *p* value was <0.05.

## 3. Results

### 3.1. NleF Inhibits Epithelial Cell Death during EHEC Infection

**Figure 1:**
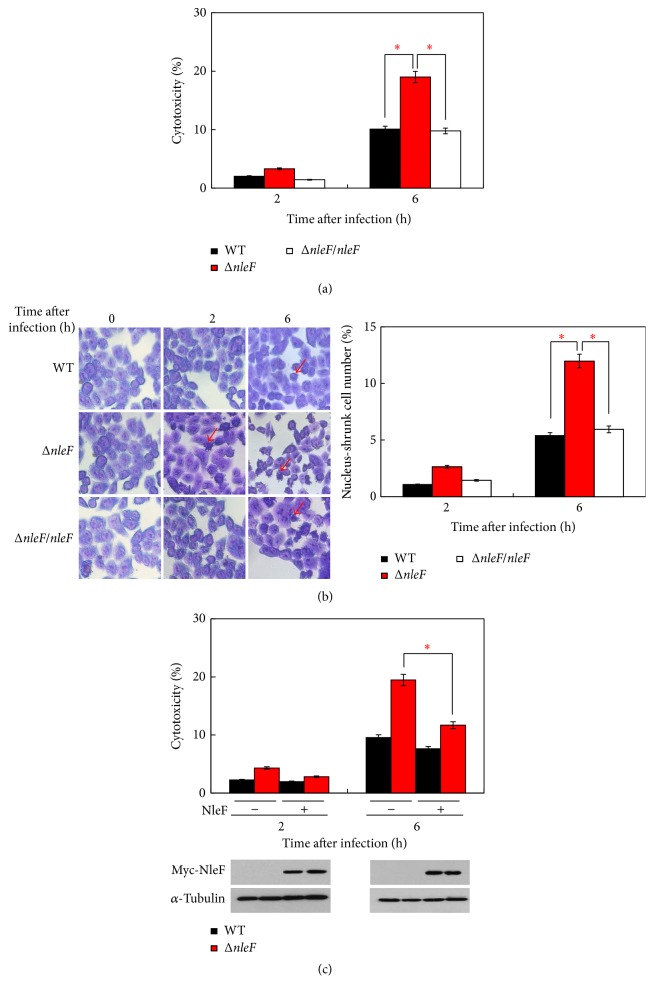
NleF inhibits epithelial cell death during EHEC infection. (a-b). HT29 cells were infected with EDL933, Δ*nleF*, or Δ*nleF/nleF* at a MOI of 100 and then incubated at 37°C for the indicated time periods. (a) Aliquots of the cellular supernatants were subjected to the cytotoxicity assay and measured at 2 and 6 hr. (b) Infected cells were fixed at the indicated time points and then subjected to Giemsa staining. Red arrowheads indicate cells whose cytoplasm disappeared. The number of infected cells undergoing chromatin condensation was calculated from at least 300 cells and is shown in the lower panels. (c) HT29 cells were transfected with Myc-vector or Myc-NleF and then were infected and subjected to cytotoxicity as (a) lysates of cells expressing Myc-NleF were analyzed by immunoblotting, and *α*-tubulin was used as an equal-loading control. Graphs show the means ± SEM of triplicate wells; all data are representative of three independent experiments. ^*∗*^*p* < 0.05. *p* values were calculated using two-tailed Student's *t*-test.

Recently, NleF has been reported to inhibit the catalytic activity of caspase-4, caspase-8, and caspase-9 [25]. It is known that caspase-4 is involved in inflammatory cell death; caspase-8 and caspase-9 are important molecules in the apoptotic pathway [26]. We speculate that the inhibition of caspases by NleF might thus also inhibit cell death. To test whether NleF inhibits cell death, we constructed the mutant lacking* nleF* (Δ*nleF*) and the* nleF*-complemented strain (Δ*nleF/nleF*). Then, we infected HT29 cells with EHEC derivatives and monitored the cell death response during bacterial infection. Based on the lactate dehydrogenase (LDH) cytotoxicity assay, the mutant lacking* nleF* (Δ*nleF*) greatly enhanced cell death at 2 and 6 hr compared to EDL933 (wild-type [WT] EHEC), whereas the* nleF*-complemented strain (Δ*nleF/nleF*) decreased the cytotoxicity to a level comparable with EDL933 (Figure 1(a)), suggesting that NleF suppresses the cell death response to EHEC infection. To confirm this finding, HT29 cells infected with different EHEC strains were subjected to Giemsa staining. As shown in Figure 1(b), Δ*nleF*-infected cells exhibited nuclear chromatin condensation at 2 and 6 hr after infection, while the rate of chromatin condensation positive EDL933 (or Δ*nleF/nleF*)-infected cells was marginal. Moreover, when HT29 cells expressing NleF were infected with Δ*nleF*, cell death was suppressed at 2 and 6 hr after infection (Figure 1(c)), confirming that NleF dampens EHEC-induced cell death. Together, these results clearly demonstrate that NleF helps counteract epithelial cell death responses to EHEC infection.

### 3.2. Characterization of EHEC Δ*nleF*-Induced Cell Death

**Figure 2:**
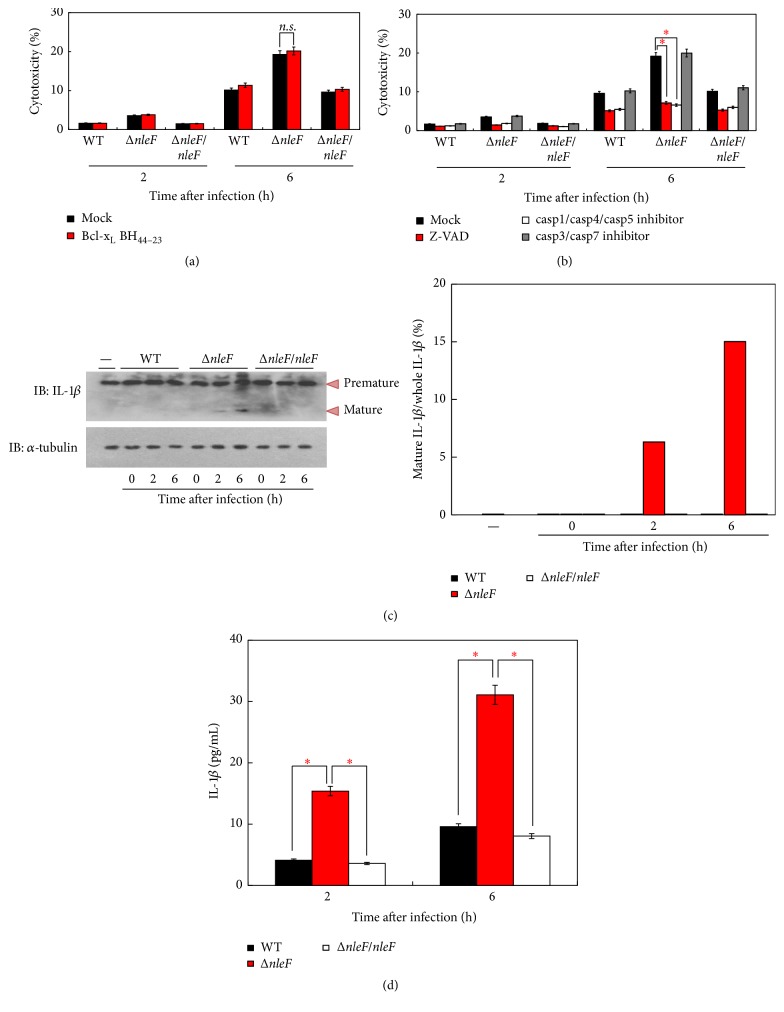
Characterization of EHEC Δ*nleF*-induced cell death. HT29 cells were infected with EDL933, Δ*nleF*, or Δ*nleF/nleF* at a MOI of 100 and then incubated at 37°C for the indicated time periods. (a-b) Infected cells were treated with or without (a) cytochrome c release inhibitor Bcl-x_L_  BH_44–23_ (1 *μ*M) or (b) caspases inhibitors (20 *μ*M). Aliquots of the cellular supernatants were subjected to the cytotoxicity assay. (c) Whole cell lysates were analyzed by immunoblotting with anti-IL-1*β* antibody; percentages of mature IL-1*β* per whole IL-1*β* are shown in the right panel. (d) Aliquots of supernatants were subjected to an IL-1*β* ELISA assay. Graphs show the means ± SEM of triplicate wells; all data are representative of three independent experiments. ^*∗*^*p* < 0.05. *p* values were calculated using two-tailed Student's *t*-test.

To further characterize the modality of EHEC Δ*nleF*-induced cell death, we treated the EDL933 (Δ*nleF* or Δ*nleF/nleF*)-infected HT29 cells with Bcl-x_L_  BH_44–23_, an inhibitor of cytochrome c release, and then examined the cell death response by LDH cytotoxicity assay. We found that Bcl-x_L_  BH_44–23_ failed to block Δ*nleF*-mediated cell death (Figure 2(a)), indicating that EHEC infection caused nonapoptotic cell death. To confirm this finding, we treated the infected cells with a pan-caspase inhibitor (Z-VAD), casp1/casp4/casp5, or casp3/casp7 inhibitor, respectively. As we expected, Δ*nleF*-induced cytotoxicity was rescued by Z-VAD and the casp1/casp4/casp5 inhibitor but not by the casp3/casp7 inhibitor (Figure 2(b)), suggesting that the modality of cell death that Δ*nleF* induced might be inflammatory cell death. Pallett et al. reported that NleF inhibited IL-18 processing during EPEC infection [22]. In this context, we examined interleukin-1*β* (IL-1*β*) maturation in cells infected with EHEC derivatives (Figure 2(c)). Immunoblot analysis revealed that IL-1*β* maturation occurred in response to Δ*nleF*, but not EDL933 (or Δ*nleF/nleF*) infection, suggesting that Δ*nleF* infection induced pyroptosis-like cell death. Furthermore, ELISA assays revealed that mature IL-1*β* was detected in Δ*nleF*-infected cells, while it was only minimally detected in EDL933 (or Δ*nleF/nleF*)-infected cells (Figure 2(d)). Together, these results indicate that casp1/casp4/casp5-mediated inflammatory cell death occurs during Δ*nleF* infection.

### 3.3. EHEC Δ*nleF*-Induced Inflammatory Cell Death Is Mediated by Caspase-4

**Figure 3:**
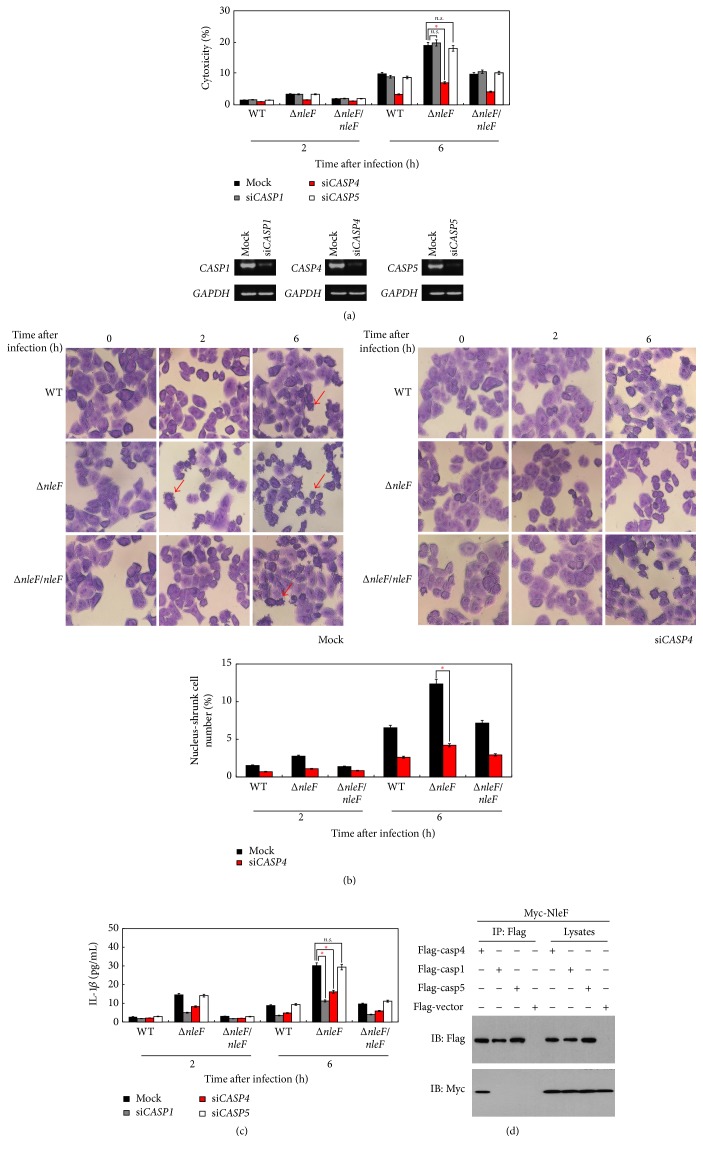
EHEC Δ*nleF*-induced inflammatory cell death is mediated by caspase-4. (a–c) HT29 cells transfected with the indicated siRNAs were infected with EDL933, Δ*nleF*, or Δ*nleF/nleF* at a MOI of 100 and then incubated at 37°C for the indicated time periods. (a) Aliquots of supernatants were subjected to the cytotoxicity assay. The knockdown efficiency of si*CASP1*, si*CASP4*, or si*CASP5* was analyzed by RT-PCR. (b) Infected cells were fixed at the indicated time points and then subjected to Giemsa staining. Red arrowheads indicate cells whose cytoplasm disappeared. The number of infected cells undergoing chromatin condensation was calculated from at least 300 cells and is shown in the lower panels. (c) Aliquots of supernatants were subjected to an IL-1*β* ELISA assay. (d) 293T cells were cotransfected with Myc-NleF and Flag-caspase-4 or Flag-caspase-1 or Flag-caspase-5 expression plasmids or Flag-vector; anti-Flag immunoprecipitates were analyzed by immunoblotting with anti-Myc or anti-Flag antibodies. Graphs show the means ± SEM of triplicate wells; all data are representative of three independent experiments. ^*∗*^*p* < 0.05. *p* values were calculated using two-tailed Student's *t*-test.

To investigate which caspase is involved in Δ*nleF*-induced inflammatory cell death, we used small interfering RNA (siRNA) to knock down caspase-1, caspase-4, or caspase-5 in HT29 cells and then infected the cells with EDL933, Δ*nleF*, or Δ*nleF/nleF*. The cytotoxicity assay showed that only caspase-4 knockdown restored Δ*nleF*-induced cell death (Figure 3(a)), indicating that EHEC Δ*nleF*-induced inflammatory cell death might be mediated by caspase-4. To confirm this finding, we examined the nuclear chromatin morphology of caspase-4 knockdown cells infected with different EHEC strains by using Giemsa staining. As shown in Figure 3(b), upon Δ*nleF* infection, cells transfected with control siRNA exhibited nuclear chromatin condensation at 2 and 6 hr after infection (2.77% and 12.36% at 2 and 6 hr, resp.), while the rate of chromatin condensation positive caspase-4 knockdown cells decreased. Then, we analyzed IL-1*β* maturation in different caspase knockdown cells infected with EHEC derivatives (Figure 3(c)). ELISA assays revealed that the secretion of mature IL-1*β* was diminished both in caspase-1 and in caspase-4 knockdown cells, suggesting that caspase-1 and caspase-4 are involved in EHEC Δ*nleF*-induced IL-1*β* secretion. Moreover, we performed coimmunoprecipitation experiment using lysates of 293T cells expressing Myc-NleF cotransfected with human Flag-caspase-1, Flag-caspase-4, or Flag-caspase-5 (Figure 3(d)). Myc-NleF was only detected in the anti-Flag immunoprecipitation from cells cotransfected with Flag-caspase-4. Based on these results, we conclude that NleF-mediated targeting of caspase-4 plays an essential role in inhibiting inflammatory cell death during EHEC infection.

### 3.4. NleF Inhibits Caspase-4 Activity by Interrupting p19-p10 Interaction

**Figure 4:**
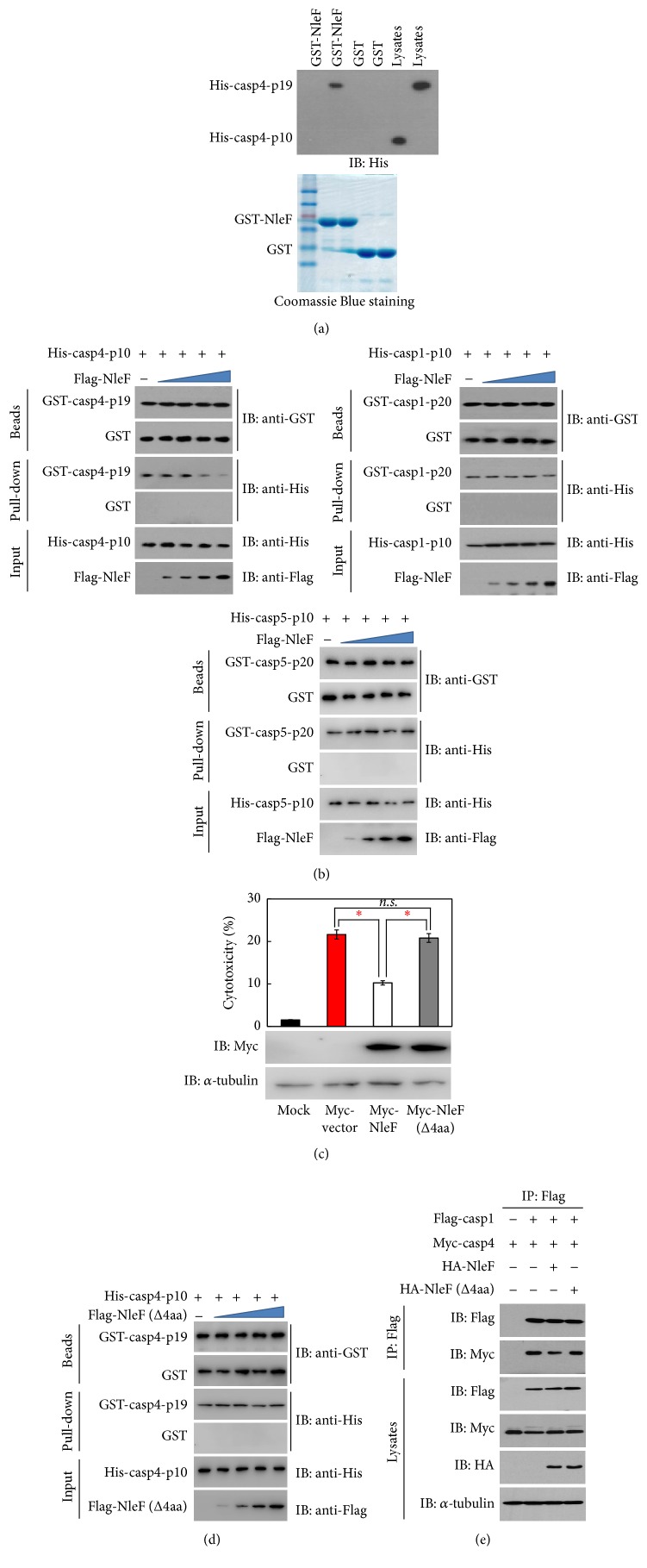
NleF inhibits caspase-4 activity by interrupting p19-p10 interaction. (a) GST-NleF or GST beads were mixed with lysates from* E. coli* cells expressing His-casp4-p19 or His-casp4-p10; the bound proteins were analyzed by immunoblotting with anti-His antibody. (b) Indicated purified proteins and GST-casp4-p19, or GST-casp1-p20 or GST-casp5-p20, were subjected to a competition assay. All bound proteins were analyzed by immunoblotting. (c) HT29 cells were transfected with Flag-casp4-p19 and Flag-casp4-p10 with Myc-NleF or Myc-NleF (Δ4aa). Supernatant aliquots were subjected to the cytotoxicity assay. Lysates of cells expressing Myc-NleF or Myc-NleF (Δ4aa) were analyzed by immunoblotting, and *α*-tubulin was used as an equal-loading control. (d) Purified His-casp4-p10, Flag-NleF (Δ4aa), and GST-casp4-p19 were subjected to a competition assay. All bound proteins were analyzed by immunoblotting. (e) 293T cells were cotransfected with Flag-caspase-1 and Myc-caspase-4 with HA-NleF or HA-NleF (Δ4aa); anti-Flag immunoprecipitates were analyzed by immunoblotting with anti-Myc or anti-Flag antibodies. Graphs show the means ± SEM of triplicate wells; all data are representative of three independent experiments. ^*∗*^*p* < 0.05. *p* values were calculated using two-tailed Student's *t*-test.

Caspase-4 exists as a precursor that consists of the caspase recruitment domain (CARD), the big catalytic subunit p19 domain, and the small catalytic subunit p10 domain. Upon activation, caspase-4 is cleaved and forms heterodimers that contain two p19 and two p10 domains [27]. To map the NleF binding domain in caspase-4, we performed a GST pull-down assay using GST-NleF, His-casp4-p19, and His-casp4-p10. As shown in Figure 4(a), only His-casp4-p19 was pulled down by GST-NleF. To investigate the effects of NleF on p19 and p10 tetramer formation, we incubated purified His-casp4-p10 with or without purified Flag-NleF and then used GST-casp4-p19 beads to pull down p10. The amount of p19 interacting with p10 was measured by immunoblotting (Figure 4(b)). As the amount of NleF increased, the binding of p19 and p10 decreased. Under the same conditions, NleF neither interfered with the interaction of the caspase-1-p20 and caspase-1-p10 nor interfered with the interaction of the caspase-5-p20 and caspase-5-p10. One recent study indicated that NleF that lacks the last four amino acids of the carboxy terminus lost the ability to interact with caspases [25]. We examined the impacts of NleF (Δ4aa) on caspase-4-dependent cell death (Figure 4(c)). Compared with WT NleF, NleF (Δ4aa) failed to prevent caspase-4-induced cell death. We next investigated the effects of NleF (Δ4aa) on p19 and p10 tetramer formation. As shown in Figure 4(d), the interaction of p19 and p10 was not affected with increasing amounts of NleF (Δ4aa) mutant. It has been reported that caspase-4 interacts with caspase-1, supporting caspase-1 processing as well as caspase-1-dependent activation of pro-IL-1*β* [28]. Our study showed that knockdown of caspase-4 could also inhibit EHEC Δ*nleF*-induced IL-1*β* secretion. We speculated that NleF might prevent the interaction of caspase-1 and caspase-4. To examine the effect of NleF on the interaction of caspase-1 and caspase-4, we cotransfected Flag-caspase-1 and Myc-caspase-4 into 293T cells with or without HA-NleF or HA-NleF (Δ4aa), prepared anti-Flag immunoprecipitates, and measured the amount of caspase-1 binding to caspase-4 (Figure 4(e)). Immunoblot analysis showed that the interaction of caspase-1 and caspase-4 was prevented by NleF but not NleF (Δ4aa). Together, these results suggest that NleF specifically interferes with caspase-4 activation via inhibiting p19 and p10 tetramer formation; the last four amino acids of the NleF carboxy terminus are essential for inhibiting caspase-4-induced cell death.

## 4. Discussion

Inflammatory cell death, which is one of the most intrinsic immune defense mechanisms of multicellular organisms, is crucial for sacrificing infected cells and controlling microbial infections. Many bacterial pathogens use distinctive strategies to modulate inflammatory cell death, which is an important pathogenic mechanism to promote replication and to avoid the release of inflammatory intracellular contents during infection [4].

Our current study revealed that NleF from EHEC could inhibit epithelial inflammatory cell death. NleF is found in the* E. coli* lineages associated with epidemic potential [29] and with human disease [30]. It is present with 100% amino acid identity in other EHEC and EPEC, and an ortholog is also found in* C. rodentium*, with which it shares 85% identity over 189 residues [23]. Blasche et al. indicated that NleF was implicated in the inhibition of apoptosis stimulated by both the intrinsic and the extrinsic pathways, but the role of NleF in apoptosis inhibition during infection remained to be determined. They observed no significant differences in the activation of downstream executioner caspase-3 and caspase-7 in HeLa cells infected with wild-type EPEC or a Δ*nleF* EPEC derivative [25]. Our experiment showed that EHEC Δ*nleF*-induced HT29 cytotoxicity was rescued by the casp1/casp4/casp5 inhibitor but not by the casp3/casp7 inhibitor (Figure 2(b)), which was consistent with Blasche's findings.

By using specific siRNA to knock down the expression of caspase-1 or caspase-4 or caspase-5, we further demonstrated that NleF prevented inflammatory cell death during EHEC infection by targeting caspase-4 (Figure 3(a)). However, caspase-1 and caspase-5, the other two essential molecules of inflammasome pathway, are not involved in this process. Pallett et al. reported that NleF could inhibit EPEC-induced caspase-4 activation and IL-18 processing, but they found no significant difference between WT EPEC and Δ*nleF* EPEC in inducing cytotoxicity of control or caspase-4-depleted Caco-2 cells [22]. This is likely due to the different cell lines and bacterial strains we used. In addition, the durations of infection in our two studies are also different.

Human caspase-4 is considered as the functional orthologs of murine caspase-11 [31]. It has been indicated that caspase-4 has a critical function in innate immunity. Knodler et al. reported that caspase-4 and caspase-11 triggered enteric bacteria induced noncanonical inflammasome activation and mature IL-18 release, as well as pyroptotic epithelial cell death and shedding. Specifically, they found that the action of these inflammatory caspases limited pathogen colonization of the intestinal epithelium [10]. Casson et al. found that caspase-4 mediated noncanonical inflammasome responses against several Gram-negative bacterial pathogens [32]. Shi et al. described an unprecedented model for caspase activation whereby caspase-4, caspase-5, and caspase-11 directly bind cytosolic LPS, triggering autoactivation and subsequent pyroptotic cell death [33]. Therefore, it is worth noting that caspase-4 might be a host target for some Gram-negative bacteria. Here, we found that NleF not only interrupted the heterodimerization of caspase-4-p19 and caspase-4-p10, but also inhibited the interaction of caspase-1 and caspase-4. The heterodimerization of p19 and p10 is essential for caspase-4 activation [27]. A previous study showed that the interaction of caspase-1 and caspase-4 promoted caspase-1-dependent activation of pro-IL-1*β* [28]. Our findings might explain the mechanism by which NleF inhibits epithelial inflammatory cell death and IL-1*β* production.

Collectively, our discovery of a bacterial inhibitor of caspase-4, which is encoded by EHEC NleF, will suggest a new way of thinking about colitis treatment and provide avenues to study EHEC pathogenesis and resulting inflammatory diseases. Our results showed that the inhibition of NleF on caspase-4-dependent inflammatory cell death might represent a potent mechanism for bacteria escaping the host innate immune response.
